# A Pilot Project to Evaluate the Acceptability of Serum Clozapine Level Monitoring by Finger-prick Method in an Adult Community Mental Health Team (CMHT)

**DOI:** 10.1192/bjo.2025.10492

**Published:** 2025-06-20

**Authors:** Sadia Tabassum Javaid, Ravindra Belgamwar, George Horton

**Affiliations:** North Staffordshire Combined Healthcare NHS Trust, Stoke-on-Trent, United Kingdom

## Abstract

**Aims:** Clozapine is a cornerstone in treating resistant schizophrenia, with evidence linking threshold plasma levels to positive clinical outcomes. The Maudsley Guidelines recommend levels of 250–420 µg/L for an adequate trial. Our trust requires trained staff to collect venous samples, with results taking several days. The updated clozapine pathway suggests checking levels at initiation and 12 months, prompting consideration of the Saladax MyCare Insite Analyzer. This point-of-care device uses a finger-prick sample and delivers results in 7 minutes. Despite promising studies, its effectiveness depends on local acceptance, warranting a pilot.

Aims were:

To evaluate the acceptability of the finger-prick test among patients and staff.

To enhance patient care with a quicker method, enabling timely referrals and decisions.

**Methods:** Clozapine testing was conducted at Lyme Brook Clozapine Clinic (23.10.2023–17.11.2023) using the Saladax MyCare Insite Analyzer on pin-prick samples, with prior patient consent and staff training. Separate anonymised questionnaires were provided for staff and patients.

**Results:** Patients (n=31) were aged 21–70 years (64.5% male, 35.5% female), majority follow ups (64.5%).

96.9% of tests were completed within 15 minutes (32.3% within 0–5 min, 58.1% within 5–10 min, 6.5% within 10–15 min, 3.2% within 15–20 min). Compared with venous blood tests, 87.1% had a positive experience, 90.3% were satisfied with the test and care received, and 90.4% valued the time-saving benefits. Overall, the test was acceptable to 90.3%, and 71.0% preferred the finger-prick test (22.6% unsure) over venous, with 90.3% willing to use it again (6.5% unsure), and 83.9% would recommend it (9.7% unsure). 97% reported no issues, with only one instance of test repetition.

Staff (n=32) were aged 31–64: 56.3% doctors, 6.3% nurse associates, 12.5% STRs, 25% trainee nurse associates; 68.8% female, 28.1% male.

96.9% completed testing within 15 minutes (0–5 min: 9.4%, 5–10 min: 62.5%, 10–15 min: 25%, 3.1% unspecified). 96.9% highly rated their experience (3.1% no response), while 100% valued time efficiency, ease of use, and care quality. 93.8% found it acceptable (6.3% neutral). 94% reported no issues, with one test repetition. All staff preferred the finger-prick test and wished to continue using it.

**Conclusion:** The pilot project showed strong acceptability of the finger-prick method among patients and staff, with high satisfaction, minimal issues, and improved time efficiency. Both groups preferred it over venous testing, supporting its potential to improve patient care.

Further evaluation of cost-effectiveness, clozapine pathway integration, and training for wider implementation across trust is recommended.

